# Anti-Inflammatory Effects of *Weissella cibaria* SDS2.1 Against *Klebsiella pneumoniae*-Induced Mammary Gland Inflammation

**DOI:** 10.3390/ani15081139

**Published:** 2025-04-15

**Authors:** Meiyi Ren, Tianxiong Jin, Jingdi Tong, Deyuan Song, Qinna Xie, Xiaohan Li, Yan Li, Kangping Liu, Jian Gao, Mingchao Liu, Jia Cheng

**Affiliations:** 1 Department of Clinical Veterinary Medicine, College of Veterinary Medicine, Hebei Agricultural University, Baoding 071001, China; renmeiyi@pgs.hebau.edu.cn (M.R.); tianxiongjin7@163.com (T.J.); tongjingdi@pgs.hebau.edu.cn (J.T.); 20232200614@pgs.hebau.edu.cn (D.S.); 20237201621@pgs.hebau.edu.cn (Q.X.); 20232200611@pgs.hebau.edu.cn (X.L.); dyly@hebau.edu.cn (Y.L.); liukangping9431@163.com (K.L.); 2 Key Laboratory of Healthy Breeding in Dairy Cattle (Co-Construction by Ministry and Province), Ministry of Agriculture and Rural Affairs, Hebei Agricultural University, Baoding 071001, China; 3 Department of Clinical Veterinary Medicine, College of Veterinary Medicine, China Agricultural University, Beijing 100193, China; gaojian2016@cau.edu.cn

**Keywords:** bovine mastitis, *Klebsiella pneumoniae*, *Weissella cibaria* SDS2.1, anti-inflammatory

## Abstract

Dairy cows are highly susceptible to mastitis caused by *Klebsiella pneumoniae*, and treating these infections poses a challenge due to the resistance of the bacterium to common antibiotics. This study aimed to evaluate the safety of *W. cibaria* SDS2.1 and investigate its protective effects against *K. pneumoniae*-induced mastitis. The safety of *W. cibaria* SDS2.1 was assessed through comprehensive analyses, including antibiotic resistance profiling, hemolysis assays, cell cytotoxicity tests, and whole-genome sequencing. Furthermore, its ability to protect against cellular and tissue damage caused by *K. pneumoniae*-induced mastitis was evaluated using both in vitro and in vivo models. Our results revealed that *W. cibaria* SDS2.1 was non-hemolytic, non-cytotoxic, and significantly inhibited the growth of *K. pneumoniae* (*p* < 0.05). Additionally, *W. cibaria* SDS2.1 effectively reduced the adhesion and invasion of *K. pneumoniae*. In the *K. pneumoniae*-induced mouse mastitis model, *W. cibaria* SDS2.1 significantly reduced myeloperoxidase (MPO) activity, mammary tissue damage, and the expression of inflammatory cytokines (IL-6, IL-1β, and TNF-α) (*p* < 0.05). In *K. pneumoniae*-infected bovine mammary epithelial cells (bMECs), *W. cibaria* SDS2.1 significantly decreased lactate dehydrogenase (LDH) release, indicating reduced cellular damage. These findings demonstrate that *W. cibaria* SDS2.1 exhibits anti-inflammatory properties in experimental models, suggesting its potential role in mitigating *K. pneumoniae*-induced mastitis.

## Simple Summary

Klebsiella pneumoniae is one of the major pathogens causing mastitis in dairy cows, posing a threat to animal welfare and milk production. In this study, we isolated a strain of Weissella cibaria SDS2.1 from dairy farm feed and evaluated its safety and anti-inflammatory effects. Antibiotic resistance profiling, hemolysis assays, cell cytotoxicity tests, and whole-genome sequencing confirmed that this strain was safe. Using a mouse mastitis model and a bovine mammary epithelial cell infection model, we found that W. cibaria SDS2.1 effectively alleviated inflammation and mitigated tissue damage caused by K. pneumoniae. These findings provide insights into the potential role of W. cibaria SDS2.1 in reducing inflammation associated with mastitis.

## 1. Introduction

Bovine mastitis is a widespread and costly disease in dairy farms, reducing milk production and quality while posing a major challenge to the dairy industry [1]. *Klebsiella pneumoniae* is a major pathogen in bovine mastitis, causing severe udder inflammation, milk losses, and increased culling rates [2]. *K. pneumoniae* can rapidly adhere to and invade bovine mammary epithelial cells (bMECs), triggering a severe inflammatory response, such as increased levels of inflammatory cytokines (IL-6, IL-8, IL-1β, and TNF-α) [3]. In addition, it induces pathological changes, including cell membrane rupture, chromatin condensation, and mitochondrial swelling [4]. Compared with *Escherichia coli* (*E. coli*), *K. pneumoniae* causes more severe clinical symptoms of mastitis [5]. Although widely used, antibiotics have contributed to antibiotic resistance, posing a serious threat to human and animal health [6]. Additionally, *K. pneumoniae* is known for its antibiotic resistance, making it particularly difficult to treat [7]. Therefore, it is essential to develop new strategies for treating bovine mastitis.

Lactic acid bacteria (LAB) have been widely explored for their potential in treating bovine mastitis [8]. Certain LAB strains inhibit pathogens by modulating the udder microbiome, enhancing immune responses, and exerting antimicrobial effects through competitive exclusion and antimicrobial production [9,10]. Recent studies also highlight ability of LAB to regulate inflammation and promote tissue repair, making them valuable for mastitis treatment. In particular, several lactic acid bacteria, such as *Lactobacillus plantarum* KLDS, have shown good therapeutic effects in clinical trials [11,12]. Therefore, LAB represent a promising new treatment strategy for controlling bovine mastitis.

*Weissella cibaria* (*W. cibaria*) is a Gram-positive, non-spore-forming, non-motile, heterofermentative LAB with a short rod shape and catalase-negative properties. Studies show that *W. cibaria* possesses antimicrobial, antioxidant, and anti-inflammatory properties, making it a potential treatment for inflammatory diseases [13,14]. It also alleviates *E. coli* and intestinal inflammation in mice, maintains intestinal mucosal integrity, and regulates gut microbiota [15]. Beyond gut health, *W. cibaria* has immunomodulatory properties in aquatic animals, significantly enhancing the defenses of Rainbow Trout (a large, stout-bodied salmonid fish native to western North America) when added to their feed [16]. Furthermore, *W. cibaria* FbpA prevents *Staphylococcus aureus* (*S. aureus*) colonization and infection by blocking its invasion through fibronectin-binding proteins and inhibiting biofilm formation [17]. To assess its safety, *W. cibaria* SDS2.1 was evaluated through antibiotic resistance profiling, hemolysis assays, cell cytotoxicity tests, and whole-genome sequencing. These findings suggest its potential role in combating bacterial infections and inflammatory conditions, making it a promising candidate for further research. Therefore, it was hypothesized that *W. cibaria* SDS2.1 may have a protective effect against inflammatory injury in bovine mastitis.

This study primarily investigated the alleviating effect of *W. cibaria* SDS2.1 on inflammatory damage caused by *K. pneumoniae* in in vitro and in vivo models. These models were chosen because they provide valuable insights into the inflammatory mechanisms associated with mastitis. Due to practical considerations, such as the size and cost of dairy cows, *mouse models* are commonly used for preliminary studies of bovine mastitis, as they are small, cost-effective, and have well-documented immune responses [18]. Although there are physiological differences between mice and cattle, the results from the mouse model provide a critical foundation for understanding how *W. cibaria* SDS2.1 alleviates *K. pneumoniae*-induced mastitis, offering potential for further research into its use as a therapeutic agent for bovine mastitis.

## 2. Materials and Methods

### 2.1. Bacterial Isolation and Source

*W. cibaria* SDS2.1 was isolated from the feed of a dairy farm in Shandong province, China, in 2023. Single white colonies were selected and purified by streaking on de Man Rogosa Sharp (MRS) medium (AOBOX, Beijing, China) under microaerophilic conditions at 37 °C for 24 h. Finally, the bacteria from the culture medium were transferred to a test tube containing 10 mL of MRS broth (AOBOX, Beijing, China) and incubated at 28 °C for 48 h. Cultures were then stored in 25% (*w*/*v*) glycerol at −80 °C for long-term preservation [19].

*K. pneumoniae* was isolated from clinical mastitis in dairy cows [3]. *K. pneumoniae* was cultured in LB broth (AOBOX, Beijing, China) at 37 °C with shaking at 180 rpm for up to 12 h. Bacterial colony counts (colony-forming units, CFUs) were determined after three generations of culture.

### 2.2. Genomic DNA Extraction and Genome Sequencing

The genomic DNA of *W. cibaria* SDS2.1 was extracted using the EasyPure^®^ Bacteria Genomic DNA Kit (TransGen Biotech, Beijing, China), and its concentration and purity were measured using a Nanodrop ND2000 spectrophotometer (ThermoFisher Scientific, Waltham, MA, USA). For sequencing, a library was prepared using the Illumina TruSeq™ DNA Sample Prep Kit (Illumina, San Diego, CA, USA) following the protocols outlined in the Illumina TruSeq Nano DNA LT Library Preparation Guide. The quality of the constructed library was evaluated using an Agilent Bioanalyzer 2100 (Agilent Technologies, Santa Clara, CA, USA), and the library concentration was precisely quantified using a Qubit Fluorometer (Thermo Fisher Scientific, Waltham, MA, USA). Whole-genome sequencing was performed on the Ion PromethION 48 System using a PromethION Flow Cell chip (Oxford Nanopore Technologies, Oxford, UK).

### 2.3. De Novo Genome Assembly and Genome Annotations

The genetic composition of the organism was analyzed using advanced bioinformatics tools. First, the genome was assembled using Canu v1.5 (University of Maryland, College Park, MD, USA), followed by circularization with Onfuse v1.5.5 (University of California, San Diego, CA, USA). Gene prediction was performed using Prodigal v2.63 (University of Texas, Austin, TX, USA). Ribosomal RNA (rRNA) and transfer RNA (tRNA) molecules were identified using Infernal v1.1.3 (Washington University, St. Louis, MO, USA) and tRNAscan-SE v2.0 (University of California, Santa Cruz, CA, USA), respectively. Repetitive sequences were annotated using RepeatMasker v4.0.5 (University of Maryland, College Park, MD, USA). Gene functions were annotated using the Gene Ontology (GO) database and the Kyoto Encyclopedia of Genes and Genomes (KEGG). The Carbohydrate-Active enZymes Database (CAZy) was analyzed using specialized databases.

### 2.4. Safety Assessment of W. cibaria SDS2.1

The safety assessment criteria for *W. cibaria* SDS2.1 included its sensitivity to antibiotics, lack of hemolytic activity, and the absence of virulence genes and antibiotic resistance genes [20,21].

#### 2.4.1. Antibiotic Resistance of *W. cibaria* SDS2.1

The minimal inhibitory concentrations (MICs) of *W. cibaria* SDS2.1 were determined in triplicate using the broth microdilution method, as described previously [22]. Briefly, a single colony of *W. cibaria* SDS2.1 from the MRS agar plates was picked and suspended in MRS broth to achieve an optical density at 625 nm (OD625) of 0.16–0.2. The bacterial suspension was diluted 1:1000 in 10% MRS broth (pH 6.7) for the MIC assay. Subsequently, 100 µL aliquots of the diluted suspension were dispensed into microplate wells, followed by the addition of 100 µL antibiotic solutions at serially diluted concentrations, achieving a final volume of 200 µL per well. The plates were then incubated anaerobically at 37 °C for 48 h. The following 10 antimicrobials at various concentrations were tested: penicillin (Sigma-Aldrich, St. Louis, MO, USA), carbenicillin (Sigma-Aldrich, St. Louis, MO, USA), cefalexin (Sigma-Aldrich, St. Louis, MO, USA), ceftriaxone (Sigma-Aldrich, St. Louis, MO, USA), cefradine (Sigma-Aldrich, St. Louis, MO, USA), ceftiofur (Sigma-Aldrich, St. Louis, MO, USA), kanamycin (Sigma-Aldrich, St. Louis, MO, USA), gentamicin (Sigma-Aldrich, St. Louis, MO, USA), enrofloxacin (Sigma-Aldrich, St. Louis, MO, USA), and timicosin (Sigma-Aldrich, St. Louis, MO, USA). *Lactobacillus rhamnosus* ATCC55826 (Guangzhou Legend Biotechnology Co., Ltd., Guangzhou, China) were included as controls in each batch of broth microdilution tests. The interpretation criteria for resistance to the 10 antimicrobials were defined as the tentative microbiological cut-off values (MCOFFs) identified by the European Food Safety Agency (EFSA) (2018) [23].

#### 2.4.2. Hemolytic Activity and Cell Viability of *W. cibaria* SDS2.1

Hemolytic activity was evaluated by culturing bacteria on Tryptic Soy Agar (TSA) plates (Qingdao High-tech Industrial Park Haibo Biotechnology Co., Ltd., Qingdao, China) supplemented with 5% sheep blood. *W. cibaria* were cultured aerobically to assess hemolysis activity using the streak plate method at 37 °C for 48 h [24]. Strains that displayed clear zones around the colonies were classified as having β-hemolysis properties. Specifically, β-hemolysis was defined as the complete lysis of red blood cells, resulting in a clear, transparent zone surrounding the colonies. The diameter of the clear zones were measured, and strains with a clear zone diameter of 2–4 mm were classified as β-hemolytic. *S. aureus* CVCC186158 (Guangzhou Legend Biotech Co., Ltd., Guangzhou, China) was used as the positive control.

The cytotoxicity of *W. cibaria* SDS2.1 was analyzed using CCK-8 kit (Solarbio Life Sciences, Beijing, China). *W. cibaria* SDS2.1 were cultured in MRS broth under aerobic conditions 37 °C for 24 h. The supernatant was centrifuged, and the bacteria were rinsed twice with sterile phosphate-buffered saline (PBS). *W. cibaria* SDS2.1 were re-suspended in serum-free DMEM/F12 base medium and adjusted to concentrations of 1 × 10^5^, 1 × 10^6^, and 1 × 10^7^ CFU/mL. The bMECs were plated in 96-well plates and incubated with different concentrations of *W. cibaria* SDS2.1 at 37 °C. Subsequently, the supernatant was sucked out and each blank was cleaned twice with PBS. A mixture of 100 μL of complete medium and 10 μL of CCK-8 reagent was added to each well and cells were incubated at 37 °C for 2 h. The absorbances at 450 nm were measured by an enzyme-labeled instrument. Cell viability was calculated according to the following formula [25]:

$$\mathrm{Cell}\;\mathrm{viability}\left(\%\right)=\frac{A\;\left(\mathrm{sample}\right)-A\;\left(\mathrm{blank}\right)}{A\;\left(\mathrm{untreated}\right)-A\;\left(blank\right)}\times 100\%$$

where A (sample) is the absorbance of the *W. cibaria* SDS2.1-treated cells, A (untreated) is the absorbance of the control cells, A (blank) is the absorbance of the complete medium.

#### 2.4.3. Virulence Genes and Antibiotic Resistance Genes of *W. cibaria* SDS2.1

The safety of *W. cibaria* SDS2.1 was evaluated by confirming the presence of virulence factors in the genome. Virulence factors were identified using the VirulenceFinder v2.0 tool (https://cge.food.dtu.dk/services/VirulenceFinder/; accessed on 28 May 2024) [26]. The genome sequence of *W. cibaria* SDS2.1 was used as the input. *Enterococcus* species were selected as references for predicting virulence factors, with a nucleotide match threshold of 90% and minimum sequence overlap of 60% between the virulence genes in the database and the *W. cibaria* SDS2.1 genome. The virulence-related genes—including those encoding toxins, hemolysins, verocytotoxin 1 (*vtx1*), verocytotoxin 2 (*vtx2*), intimin (*eae*), and verocytotoxin (*vtx*)—were analyzed.

For the genomic analysis of *W. cibaria* SDS2.1, the antibiotic resistance genes in the *W. cibaria* SDS2.1 genome were screened using the CARD (Comprehensive Antibiotic Resistance Database, https://card.mcmaster.ca/; accessed on 28 May 2024) [27] with thresholds of >80% identity and >60% coverage. After that, the mobile genetic elements in the genome were searched to determine the possibility of horizontal gene transfer.

### 2.5. Antibacterial Activity of W. cibaria SDS2.1

The antibacterial activity of *W. cibaria* SDS2.1 against *K. pneumoniae* was assessed using a co-culture method [28]. The concentration of a *K. pneumoniae* and *W. cibaria* SDS2.1 bacteria solution was adjusted to 1 × 10^9^ CFU/mL, and 100 μL was absorbed and introduced to 5 mL of the MRS broth medium, whereas a separate culture of *K. pneumoniae* served as the control. The viable count of *K. pneumoniae* in the co-culture system was measured after incubation at 37 °C with shaking at 180 rpm for 0 h, 12 h, and 24 h. MacConkey inositol adonitol carbenicillin agar (MCIC) plates (Qingdao High-tech Industrial Park Haibo Biotechnology Co., Ltd., Qingdao, China) were used to enumerate the bacteria, and the differences in viable counts were compared to those of the control group (Table 1).

### 2.6. Acid Production Capacity of W. cibaria SDS2.1

*W. cibaria* SDS2.1 was cultured in MRS broth at 37 °C for 24 h. The pH of the bacterial culture was monitored every 2 h for 24 h using a FiveEasystandard pH meter (Mettler Toledo, Columbus, OH, USA). The average values from three consecutive samples were used to plot the acid production curves.

### 2.7. Antibacterial Activity Under Different pH Conditions

To investigate the antibacterial effects of *W. cibaria* SDS2.1 under different pH conditions, the pH of the MRS broth medium was adjusted to values ranging from 4 to 7 (4, 4.5, 5.0, 5.5, 6.0, 6.5, 7.0). *W. cibaria* SDS2.1 was inoculated into the MRS broth media at the adjusted pH values. The antibacterial effect of *W. cibaria* SDS2.1 under different pH conditions was measured using the Oxford cup method. The liquid culture of *W. cibaria* SDS2.1 was centrifuged at 14,600× *g* for 15 min at 4 °C and filtered through 0.22 μm membranes to obtain a bacteria-free supernatant. *K. pneumoniae* was adjusted to 0.5 McFarland (1 × 10^9^ CFU/mL), and 100 μL was plated on Mueller–Hinton agar (MHA) (Qingdao High-tech Industrial Park Haibo Biotechnology Co., Ltd., Qingdao, China). The Oxford cups were placed evenly on the plate and 200 μL of bacteria-free supernatant was added to the Oxford cups, while the untreated MRS broth medium served as the negative control. The diameter of the inhibition zones was measured after incubation at 37 °C for 24 h.

### 2.8. The Impact of pH on the Growth of W. cibaria SDS2.1

To investigate the growth of *W. cibaria* SDS2.1 under different pH conditions, the pH of the MRS broth medium was adjusted to values ranging from 4.0 to 7.0. *W. cibaria* SDS2.1 was inoculated into the adjusted MRS broth media, and all the cultures were incubated anaerobically at 37 °C for 24 h. The growth of bacteria was monitored by measuring the absorbance of the bacterial fluid at 600 nm every 2 h (2, 4, 6, 8, 10, 12, 14, 16, 18, 20, 22, and 24 h).

### 2.9. Bovine Mammary Epithelial Cell (bMEC) Culture and Treatment

The bMECs (MAC-T line) were obtained from Shanghai Jingma Biological Technology (Shanghai, China). The bMECs were cultured in 89% DMEM/F12 medium (Procell, Wuhan, China) supplemented with 10% heat-inactivated fetal bovine serum (FBS) (MeilunBio, Dalian, China) and 1% penicillin–streptomycin solution (Solarbio, Beijing, China) in cell culture plates (Corning Inc., Corning, NY, USA). The bMECs were cultured to logarithmic growth and then inoculated into 6-well or 96-well plates until the desired confluence was obtained (about 90%); the cells were washed twice with PBS for the subsequent experiments. The culturing environment was strictly controlled at a temperature of 37 °C with 5% CO_2_.

**Figure 1:**
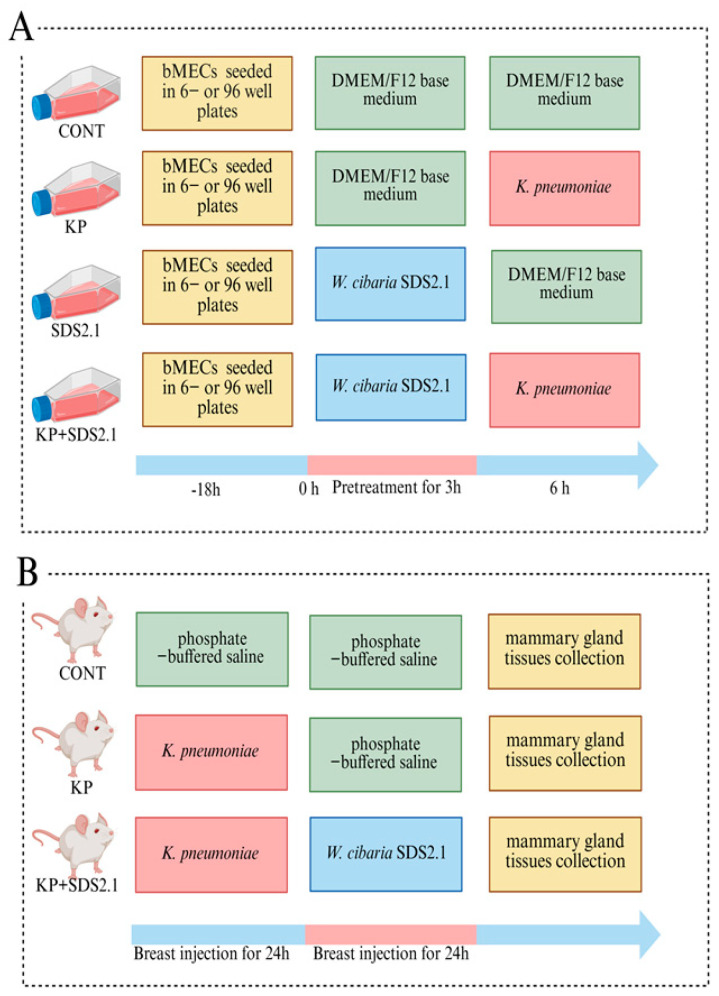
Experimental design. (**A**) In vitro experimental design. (**B**) In vivo experimental design.

The cells were divided into four groups: the control group (medium only); the *K. pneumoniae* infected group (bMECs were infected with *K. pneumoniae* at a multiplicity of infection (MOI) of 5); the *W. cibaria* SDS2.1 control group (medium only containing 1 × 10^6^ CFU/mL *W. cibaria* SDS2.1); and the *W. cibaria* SDS2.1 + *K. pneumoniae* group (the bMECs were pretreated with *W. cibaria* SDS2.1 for 3 h and then infected with *K. pneumonia* for 6 h). Each group had three replicate wells (Figure 1A).

### 2.10. Adhesion and Invasion

Adhesion and invasion were measured following the method described by Cheng [3]. *W. cibaria* SDS2.1 was added to bMECs for 3 h, followed by treatment with *K. pneumoniae* for 3, 6, and 9 h. After incubation, the bMECs were washed with PBS and treated with trypsin. The cell suspension was diluted with DMEM/F12 medium and then cultured on an MCIC plate to determine the number of bacteria adhering to the surface of the bMECs. To evaluate the number of *K. pneumoniae* invading the bMECs, the cells were washed with PBS and treated with 1 mL of DMEM/F12 containing kanamycin (100 µg/mL) (Solarbio, Beijing, China) for 2 h. After that, the cells were lysed with 0.5% Triton X-100 (Solarbio, Beijing, China). The lysate was diluted and cultured on an MCIC plate as described above. The colonies were counted, and the invasion rate of *K. pneumoniae* into the bMECs was calculated.

### 2.11. Animals and Experimental Design

SPF-grade male and female KM mice (6–8 weeks old) were purchased from Specific Pathogen Free (SPF) Biotechnology Co. (Beijing, China). These mice were selected for their high fecundity and maternal adaptability, important for lactation studies [29]. Additionally, this outbred strain has been widely used in infectious disease models in China, providing comparable baseline data [30].

After 7 days of acclimatization, female mice were paired with male mice at a 3:1 ratio for mating. After natural mating and pregnancy, the female mice were housed alone in cages at the Experimental Animal Center of Hebei Agricultural University, where they were regularly fed and had free access to water.

Eighteen pregnant mice were randomly divided into three groups (n = 6): a control group (CONT), *K. pneumoniae* infection group (KP), and *W. cibaria* SDS2.1 treatment group (KP + SDS2.1). On the 7th day after delivery, the pups were separated from the mothers, and the experiment began. After anesthetizing the female mice with Zoletil 50 (Virbac, Carros, France; 0.05 mg/kg i.m.), the fourth pair of nipples were disinfected with 75% alcohol. With the aid of a stereomicroscope, liquid was slowly injected into the mammary gland with a micro-syringe (Hamilton, Reno, NV, USA) and a 33-gauge blunt needle. In the KP and KP + SDS2.1 groups, each mammary gland was injected with 100 μL of *K. pneumoniae* suspension (1 × 10^4^ CFU/mL), while the CONT group received 100 μL PBS. After 24 h, *W. cibaria* SDS2.1 (1 × 10^6^ CFU/mL) was injected into the KP + SDS2.1 group, and the other groups received PBS. The *K. pneumoniae* dosage was adjusted from previous studies to induce mastitis without harming the animals [31]. The *W. cibaria* SDS2.1 dosage was based on cell viability tests to ensure safety. After another 24 h, the overall condition of the mice was evaluated by two independent researchers, and Cohen’s Kappa (κ = 0.85) was used to evaluate inter-rater reliability, indicating strong consistency (Table 2) [32]. The mice were euthanized using pentobarbital sodium (Sigma-Aldrich, St. Louis, MO, USA; 100 mg/kg i.m.), and the mammary tissues were excised under sterile conditions. Tissues were either stored at −80 °C for further experiments or fixed in 4% paraformaldehyde solution (Solarbio, Beijing, China) for histological analysis (Figure 1B).

### 2.12. Enumeration of K. pneumoniae in Mammary Gland

Mammary gland tissue was collected under sterile conditions. A tissue homogenizer (Omni International, Kennesaw, GA, USA) was used, and 1 mL of PBS was used to homogenize 0.1 g of the mammary gland. The tissue homogenate was diluted with sterile physiological saline and the diluent was spread on MCIC. The culture was inverted at 37 °C for 16 h in a constant-temperature biochemical incubator (Thermo Fisher Scientific, Waltham, MA, USA). We counted the number of *K. pneumoniae* colonies on the agar plate and calculated the quantities of *K. pneumoniae* in the mammary gland based on the colony count.

### 2.13. Histopathological Observations

The mammary tissue samples were fixed in a 4% paraformaldehyde solution, dehydrated through a graded alcohol series, cleared with xylene, and embedded in paraffin. Subsequently, the paraffin-embedded tissues were sectioned into 5 μm slices, stained routinely with hematoxylin and eosin (H&E) (Biyuntian Biotechnology Co., Ltd., Shanghai, China), and observed under a light microscope (Nikon, Tokyo, Japan).

The H&E-stained tissues were also examined using a light microscope at 100× and 400× magnification to evaluate the degree of inflammatory cell infiltration in the mammary tissue. Each tissue section was analyzed in triplicate to ensure a representative sample of the tissue’s inflammatory response. The average number of inflammatory cells per section was calculated, and statistical comparisons were performed between the experimental groups.

### 2.14. Myeloperoxidase Evaluation

Myeloperoxidase (MPO) activity was measured using an MPO kit (Nanjing Jiancheng Bioengineering Institute, Nanjing, China) according to the instructions of the manufacturer. Mammary gland tissues were homogenized in reaction buffer at a ratio of 1:9 (*w*/*v*) prior to the assay. MPO activity was calculated using the following formula: (ODtest-ODcontrol)/[11.3 × tissue weight (g)].

### 2.15. Transcriptional Gene Expression of Inflammatory Genes

The total RNA of the bMECs and mouse mammary tissues was extracted and the cDNA was synthesized with a TransZol Up Plus RNA Kit (TransGen Biotech, Beijing, China) and a RevertAid First Strand cDNA synthesis kit (Tiangen, Beijing, China), respectively. The mRNA levels were determined using the CFX Connect Real-Time System (Bio-Rad) with SuperReal PreMix Plus (Tiangen, Beijing, China). Primers for the target genes Interleukin (IL)-6, IL-1β, IL-8, IL-18, Tumor Necrosis Factor (TNF)-α, and the reference gene glyceraldehyde-3-phosphate dehydrogenase (GAPDH) were designed using Primer3 software (version 4.1.0) (http://primer3.ut.ee/; accessed on 1 June 2024), ensuring high specificity and efficiency [33]. The primer sequences are listed in Table 3. All the primers were synthesized by Sangon Biotech (Shanghai, China). The reaction mixture was incubated as follows: 95 °C for 15 min, followed by 40 cycles of 95 °C for 10 s and 60 °C for 30 s. The annealing temperature was 60 °C. The relative expression of mRNA was calculated using the 2^−ΔΔCT^ (cycle threshold) method to determine the expression of the related genes. The primer sequences are listed in Table 3.

### 2.16. Secreted Inflammatory Cytokines Quantification

To identify cytokines, the supernatants from the treated bMECs in 6-well plates were collected. The mammary gland tissues from the same group were homogenized, followed by centrifugation at 14,600× *g* for 20 min to collect the supernatant. The protein levels of IL-6 (minimal detectable dose is 14.22 pg/mL), IL-8 (minimal detectable dose is 20.81 pg/mL), IL-1β (minimal detectable dose is 7.03 pg/mL), TNF-α (minimal detectable dose is 18.84 pg/mL), and IL-18 (minimal detectable dose is 18.62 pg/mL) in the bMECs, as well as IL-6 (minimal detectable dose is 2 pg/mL), IL-1β (minimal detectable dose is 0.93 pg/mL), and TNF-α (minimal detectable dose is 16.85 pg/mL) protein levels in the mouse mammary gland tissues, were measured using specific ELISA kits (Shanghai Enzyme-linked Biotechnology Co., Ltd., Shanghai, China).

### 2.17. Morphology of bMECs

The bMECs were treated with *W. cibaria* SDS2.1 and *K. pneumoniae* and incubated at 37 °C with 5% CO_2_ for 3, 6, and 9 h. After incubation, the cells were washed with PBS, fixed in 4% paraformaldehyde for 20 min, washed twice with PBS, and air-dried for 10 min. H&E staining (Nanjing Jiancheng Bioengineering Institute, Nanjing, China) was performed according to the instructions of the manufacturer. The cells were dehydrated through a graded alcohol series (75%, 85%, 95%, and 100%) and observed under a light microscope at 200× magnification [35].

### 2.18. Lactate Dehydrogenase (LDH) Activity Assay

Using the LDH Cytotoxicity Assay Kit (Jiancheng Bioengineering Institute, Nanjing, China) according to the instructions of the manufacturer, the release of LDH into the culture medium due to cell damage was measured as an indicator of cytotoxicity. Briefly, after cell modeling in a 96-well plate, the supernatant was removed and LDH release reagent was added to each well. The plate was gently mixed and incubated for 1 h. After incubation, the plate was centrifuged again at 400× *g* for 5 min. A 120 μL aliquot of the supernatant from each well was transferred to a new 96-well plate, and LDH activity was quantified by measuring the absorbance at 450 nm using a microplate reader.

### 2.19. Statistical Analyses

The experimental results were derived from three independent experiments, each performed in triplicate. Values were expressed as means ± standard errors of means (SEMs). Statistical significance was calculated using one-way ANOVA, followed by the Duncan and LSD multiple tests, using SPSS 27.0 (SPSS Inc., Chicago, IL, USA). No common letter indicates a significant difference (*p* < 0.05) between treatments. GraphPad Prism 8.0 software was used for all analyses.

## 3. Results

### 3.1. Genome Annotation and Gene Ontology-Based Functional Characterization of W. cibaria SDS2.1

The circular genome map of *W. cibaria* SDS2.1 shows that its genome consists of a 2.42 Mb chromosome and a 49.95 kb plasmid, with a total size of 2.47 Mb and a G+C content of 45.02%, which is related to the genetic stability and replication process of the bacterium. The map highlights genes involved in essential metabolic processes, including carbohydrate and amino acid transport, as well as nucleotide metabolism, which are critical for the bacterium’s growth and the adaptability of *W. cibaria* SDS2.1 (Figure S1). The majority of the genes were related to metabolism, particularly carbohydrate transport and metabolism. The KEGG annotations covered three domains—environmental information processing, genetic information processing, and metabolism—with a significant number of genes involved in ABC transport, amino acid biosynthesis, and purine metabolism (Figure S2A). *W. cibaria* SDS2.1 contains genes associated with anti-inflammatory, antibacterial, and immune regulation pathways, as shown in Table S1. Notably, eight genes were annotated as being involved in ubiquinone and other terpenoid-quinone biosynthesis. This pathway primarily produces ubiquinone and other terpenoids, such as phylloquinone and vitamin K, which can inhibit inflammatory responses through their antioxidant and regulatory signaling functions. The GO annotations categorized the genes into biological processes, cellular components, and molecular functions, focusing on localization, metabolism, and cellular functions (Figure S2B). eggNOG annotations identified 1709 genes across 21 categories with significant roles in carbohydrate transport, translation, and amino acid metabolism (Figure S3A). CAZy analysis revealed 87 enzyme-encoding genes, with glycoside hydrolases and glycosyl transferases being the most abundant (Figure S3B).

### 3.2. The Safety and Antibacterial Activity of W. cibaria SDS2.1

In the hemolysis test, a clear transparent zone was observed around S. aureus, indicating hemolytic activity (Figure S4A), while no such zone was observed for W. cibaria SDS2.1 (Figure S4B), confirming that *W. cibaria* SDS2.1 is non-hemolytic. The results of the cytotoxicity test showed that co-incubating *W. cibaria* SDS2.1 with bMECs at a concentration of 1 × 10^6^ CFU/mL for 12 h had no significant effect on cell viability (Figure S4D). The sensitivity of *W. cibaria* SDS2.1 to antibiotics was tested. This strain was sensitive to most antibiotics (penicillin, carbenicillin, cefalexin, ceftriaxone, cefradine, ceftiofur, and enrofloxacin) and exhibited moderate resistance to others (kanamycin, gentamicin, and tilmicosin) (Figure S4I). The analysis of the CARD revealed no “Perfect”-category antimicrobial resistance (AMR) genes, indicating that there were no genes with complete sequence matches to known resistance determinants. However, two “Strict”-category AMR genes were identified, with sequence identities ranging from 33.33% to 34.95%. This suggests the presence of genes with partial homology or lower confidence matches to known AMR sequences. These genes, summarized in Table S2, are linked to potential resistance mechanisms but do not correspond to high-risk resistance profiles. VirulenceFinder analysis revealed no virulence factors in the genome of *W. cibaria* SDS2.1 (Figure S5).

The morphology of *W. cibaria* SDS2.1 on MRS agar medium is uniform, presenting a smooth, milky-white surface. The edges are well-defined, and the texture of the colonies is moist. The size of the colonies is uniform, and they exhibit no distinct color changes or irregularities. Under appropriate conditions, the growth is even, with no signs of translucency or pigmentation (Figure S4C). *W. cibaria* SDS2.1 demonstrated antibacterial activity against *K. pneumoniae*. When co-cultured, the number of *K. pneumoniae* significantly decreased after 12 and 24 h compared to the control group (Figure S4E). The antibacterial activity of *W. cibaria* SDS2.1 against *K. pneumoniae* under different pH conditions is shown in Figure S4F. The maximum inhibitory diameter against *K. pneumoniae* was observed at pH 6.5. The acid production capacity of *W. cibaria* SDS2.1 was analyzed, and the pH of the culture medium decreased over time. After 24 h of cultivation, the pH dropped to 3.71, indicating a strong acid-producing ability (Figure S4H). The growth of *W. cibaria* SDS2.1 under various pH levels indicates optimal growth at pH 6 to 7 (Figure S4G). *W. cibaria* SDS2.1 became progressively less able to grow as the environment became more acidic (lower pH).

### 3.3. W. cibaria SDS2.1 Alleviates K. pneumoniae-Induced Injury to In Vivo Infection Model

**Figure 2:**
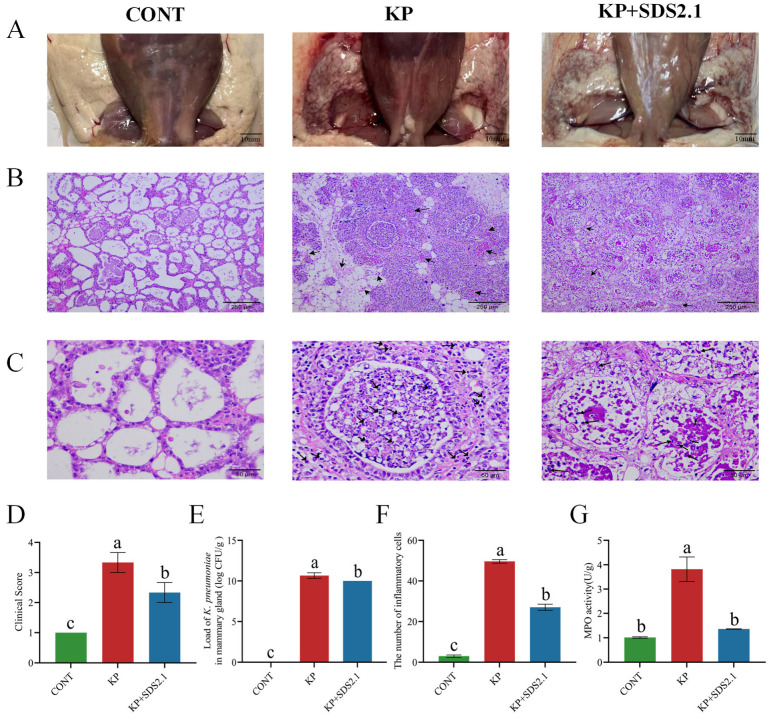
*W. cibaria* SDS2.1 alleviates *K. pneumoniae*-induced injury to mice mastitis. (**A**) Images of mammary gland tissue from mice in various treatment groups (scale bar = 10 mm). (**B**) H&E staining of mammary gland tissue from mice in different treatment groups (low-magnification, scale bar = 250 μm). (**C**) H&E staining of mammary gland tissue from mice in different treatment groups (high-magnification, scale bar = 50 μm)**.** (**D**) Mouse clinical score. (**E**) Load of *K. pneumoniae* in mammary tissue. (**F**) Inflammatory cell infiltration in mammary tissue of mice in different treatment groups. (**G**) MPO activity in mammary tissue. Data presented as mean ± SEM for three independent experiments. Different letters (a, b, c) indicate significant differences (*p* < 0.05) between treatments.

The effects of *W. cibaria* SDS2.1 on the mammary glands were investigated using a *K. pneumoniae*-induced mouse mastitis model. The observation and clinical scoring of mice in different treatment groups showed that the KP + SDS2.1 group was more active and had significantly lower clinical scores compared to the KP group (*p* < 0.05) (Figure 2D). *W. cibaria* SDS2.1 alleviated hemorrhage and redness in mammary tissue caused by *K. pneumoniae* infection (Figure 2A). As shown in Figure 2E, no bacterial colonization was observed in the control group. Compared to the KP group, the bacterial load in the *W. cibaria* SDS2.1 treatment group was significantly reduced (*p* < 0.05). H&E staining showed that the KP group exhibited significant histological changes, with abnormalities present in the mammary gland and disruption of the tissue structure. The KP + SDS2.1 group showed a reduction in these changes, indicating some beneficial effects of the treatment (Figure 2B). At higher magnification, more detailed observations of tissue damage were noted, particularly an increase in inflammatory cell infiltration in the KP group, with unclear acinar contours. In contrast, the KP + SDS2.1 group displayed fewer inflammatory cells and clearer acinar contours (Figure 2C). Additionally, the inflammatory cell count showed that the KP group had the highest number of inflammatory cells, while the KP + SDS2.1 group exhibited a significant reduction (Figure 2F), suggesting that *W. cibaria* SDS2.1 treatment can alleviate the inflammatory response. The MPO assay further indicated that treatment with *W. cibaria* SDS2.1 significantly reduced neutrophil infiltration induced by *K. pneumoniae* (*p* < 0.05) (Figure 2G).

### 3.4. W. cibaria SDS2.1 Reduced K. pneumoniae-Induced Inflammation in Mice Mastitis

**Figure 3:**
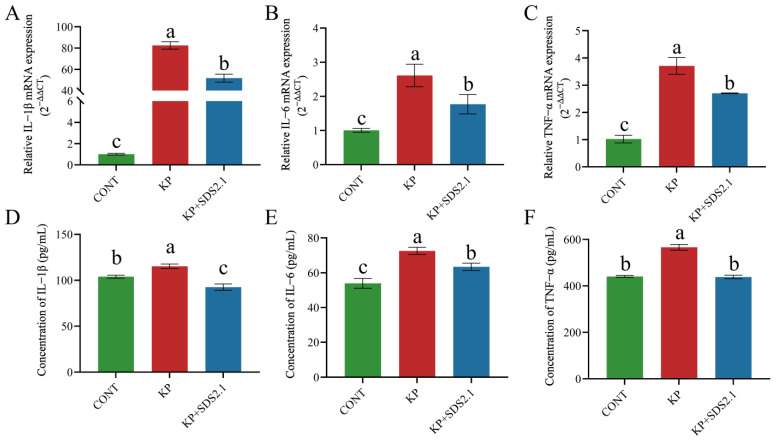
*W. cibaria* SDS2.1 reduces expression of inflammatory factors in *K. pneumoniae*-induced mouse mastitis. (**A**–**C**) Relative mRNA expression levels of TNF-α, IL-1β, and IL-6 in mouse mammary tissue homogenates, measured by qRT-PCR. (**D**–**F**) Protein levels of TNF-α, IL-1β, and IL-6 in mammary tissue homogenates, measured by ELISA. Data presented as means ± SEMs from three independent experiments. Different letters (a, b, c) indicate statistically significant differences (*p* < 0.05) between treatments.

The relative expression levels of IL-6, IL-1β, and TNF-α mRNA in the mouse mammary glands were measured by qRT-PCR. The data were normalized to the housekeeping gene GAPDH and presented as fold change relative to the control group (CONT). *K. pneumoniae* infection significantly increased the mRNA expression of inflammatory cytokines in mouse mammary glands (*p* < 0.05). KP + SDS2.1 significantly reduced the mRNA expression levels of these cytokines (*p* < 0.05) compared to *K. pneumoniae*-infected mammary glands (Figure 3A–C).

IL-1β, IL-6, and TNF-α expression levels in mammary tissues were by measured ELISA. As shown in Figure 3D–F, the expression levels of IL-1β, IL-6, and TNF-α were higher in the KP group than in the CONT group (*p* < 0.05). Additionally, the expression levels of these cytokines were lower in the KP + SDS2.1 group than in the KP group (*p* < 0.05). The results from the ELISA and qRT-PCR were consistent, indicating that *W. cibaria* SDS2.1 effectively reduced the expression of inflammatory cytokines in *K. pneumoniae*-infected mammary tissue.

### 3.5. W. cibaria SDS2.1 Reduced Pathomorphological Changes and LDH Levels in K. pneumoniae-Infected bMECs

**Figure 4:**
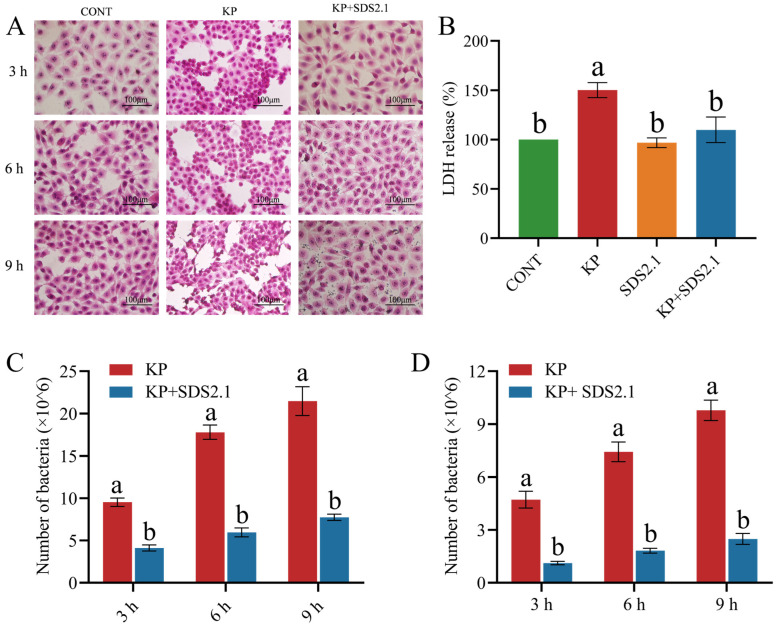
Pretreatment with *W. cibaria* SDS2.1 improved inflammation in *K. pneumoniae*-infected bMECs. (**A**) Morphological observations (scale bar = 100 μm). (**B**) LDH release. (**C**,**D**) Adhesion and invasion of *K. pneumoniae*-infected bMECs with and without pretreatment with *W. cibaria* SDS2.1. Data presented as mean ± SEM from three independent experiments. Different letters (a, b) indicate statistically significant differences (*p* < 0.05) between treatments.

Compared to the CONT group, at 3 h post-infection (hpi), the KP group exhibited blurred cell boundaries, mild detachment, edema, and nuclear hyperchromasia. Over time, the KP group showed significant cell detachment and edema. At 9 hpi, nuclear karyolysis was observed, whereas the KP + SDS2.1 group showed no significant pathological changes (Figure 4A). LDH secretion was significantly higher in *K. pneumoniae*-infected bMECs compared to the control group (*p* < 0.05), whereas pretreatment with *W. cibaria* SDS2.1 significantly reduced the LDH levels in the bMEC supernatant (*p* < 0.05) (Figure 4B).

### 3.6. W. cibaria SDS2.1 Reduced Adhesion and Invasion of K. pneumoniae to bMECs

*K. pneumoniae* heavily adhered to and invaded the bMECs between 3 and 9 h. Pretreatment with *W. cibaria* SDS2.1 significantly reduced both the adhesion and invasion of *K. pneumoniae* at all three time points (3, 6, and 9 h) (*p* < 0.05) (Figure 4C,D).

### 3.7. W. cibaria SDS2.1 Inhibited Inflammatory Responses in Infected bMECs

**Figure 5:**
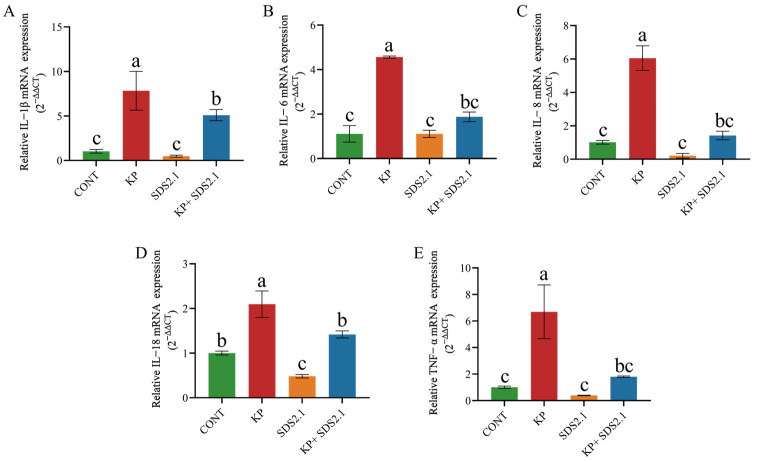
Pretreatment with *W. cibaria* SDS2.1 modulates relative mRNA expression levels of inflammatory cytokines in *K. pneumoniae*-infected bMECs. (**A**) Relative mRNA expression levels of IL-1β. (**B**) Relative mRNA expression levels of IL-6. (**C**) Relative mRNA expression levels of IL-8. (**D**) Relative mRNA expression levels of IL-18. (**E**) Relative mRNA expression levels of TNF-α. Relative mRNA expression levels of inflammatory cytokines in *K. pneumoniae*-infected bMECs measured by qRT-PCR. Data presented as mean ± SEM from three independent experiments. Different letters (a, b, c) indicate significant differences (*p* < 0.05) between treatments.

The relative expression levels of inflammatory cytokine (IL-6, IL-8, IL-1β, TNF-α, and IL-18) mRNA in bMECs were measured by qRT-PCR. The data were normalized to the housekeeping gene GAPDH and presented as fold change relative to the control group (CONT). *K. pneumoniae* infection significantly increased the mRNA expression of inflammatory cytokines (IL-6, IL-8, IL-1β, TNF-α, and IL-18) in the bMECs (*p* < 0.05). Pretreatment with *W. cibaria* SDS2.1 significantly reduced the mRNA expression levels of these cytokines (*p* < 0.05) compared to the *K. pneumoniae*-infected bMECs (Figure 5A–E).

**Figure 6:**
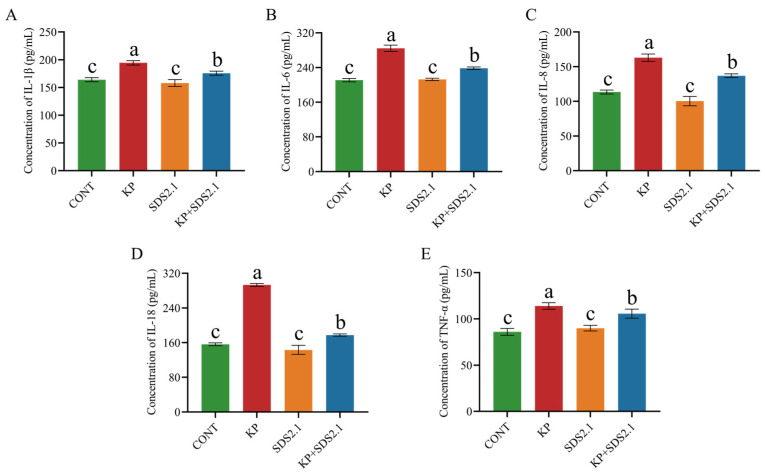
Pretreatment with *W. Cibaria* SDS2.1 modulates protein concentrations of inflammatory cytokines in *K. pneumoniae*-infected bMECs. (**A**–**E**) Concentrations of inflammatory cytokine proteins in supernatants of *K. pneumoniae*-infected bMECs with and without pretreatment with *W. cibaria* SDS2.1. Data presented as mean ± SEM from three independent experiments. Different letters (a, b, c) indicate significant differences (*p* < 0.05) between treatments.

*K. pneumoniae* infection significantly increased the secretion of IL-6, IL-8, IL-1β, TNF-α, and IL-18 in the bMECs compared to the control group (*p* < 0.05). Pretreatment with *W. cibaria* SDS2.1 significantly reduced the secretion levels of these inflammatory cytokines compared to the *K. pneumoniae*-infected bMECs (*p* < 0.05) (Figure 6A–E).

## 4. Discussion

*K. pneumoniae* is a Gram-negative environmental pathogen that can cause clinical and subclinical mastitis [36]. Clinical mastitis caused by *K. pneumoniae* is characterized by a prolonged subclinical stage, severe inflammation, and poor clinical effects [37]. *K. pneumoniae*-induced mastitis poses a great threat to cow survival and milk production [38]. However, *K. pneumoniae* has developed resistance to many commonly used antibiotics. This resistance complicates the treatment of mastitis and requires a more strategic and prudent approach to antibiotic use [6]. Recent studies have shown that certain LAB can directly suppress bovine mastitis pathogens and prevent the emergence of resistant bacteria [13,39,40]. *W. cibaria*, which lack hemolytic, cytotoxic, and inflammatory properties, emerge as ideal candidates for combating bovine mastitis due to their safe and potent antibacterial activity [41]. *W. cibaria* SDS2.1 was chosen for its safety, ability to fight *K. pneumoniae* bacteria, and anti-inflammatory properties, which could be beneficial during infection. Importantly, *W. cibaria* SDS2.1 remained active over a broad pH range (2.0–6.0), which is crucial for maintaining the effectiveness of its antimicrobial compounds. *W. cibaria* SDS2.1 showed the highest inhibitory activity at pH 6.5, a result that can be attributed to several factors. First, the weakly acidic environment at pH 6.5 may enhance the antimicrobial activity of certain compounds by altering the bacterial cell membrane’s structure and function. Second, the pH-sensitive nature of some antimicrobial peptides allows them to be more effective under slightly acidic conditions [42]. Overall, this study demonstrates that *W. cibaria* SDS2.1 is a promising candidate for the treatment of *K. pneumoniae*-induced mastitis.

Probiotic safety assessment requires evaluation factors such as antibiotic resistance, hemolytic activity, and toxin production [21]. Whole-genome sequencing (WGS) is essential for assessing probiotic safety. It provides a comprehensive DNA sequence of the organism, offering insights into bacterial genetics, evolution, and epidemiology [22]. In this study, the safety of *W. cibaria* SDS2.1 for probiotic use was assessed through phenotypic and genotypic analysis, including cytotoxicity testing, hemolysis testing, antibiotic sensitivity testing, and virulence gene analysis. *W. cibaria* SDS2.1 exhibited hemolysis-negative and non-cytotoxic properties, and did not induce any inflammation. Furthermore, the whole-genome sequencing of *W. cibaria* SDS2.1 showed no presence of virulence or antibiotic resistance genes. These characteristics demonstrate the excellent potential of *W. cibaria* SDS2.1 as a safe and effective antibacterial agent, highlighting its promise as a candidate for the treatment of bovine mastitis.

Cell viability and cytotoxicity tests play a crucial role in evaluating the cytotoxic effects of substances and can detect whether molecules have an effect on cell proliferation or show direct cytotoxic effects [43]. There are various methods for detecting cell viability and toxicity, including CCK-8, LDH, MTT, and MTS. The LDH release assay measures lactate dehydrogenase activity in the culture medium; LDH is present in all cells and is rapidly released into the cell culture supernatant when the plasma membrane is damaged, which is a key feature of apoptosis, necrosis, and other forms of cell damage [44]. The CCK-8 assay is based on the reduction of WST-8 by cellular dehydrogenases in viable cells to produce formazan. Similarly, MTT and MTS assays are also based on the reduction of tetrazolium salts by viable cells [45,46]. In our study, CCK-8 was used to detect the metabolic activity of bMECs and evaluate cell survival after treatment with *W. cibaria* SDS2.1. LDH was employed to assess the membrane integrity of bMECs, providing additional insights into cytotoxic effects. The CCK-8 assay has been widely used in studies on the safety of probiotics due to its advantages of being non-toxic, requiring short detection times, and exhibiting high sensitivity. Meanwhile, the LDH release assay is commonly employed as a complementary test to evaluate cell membrane integrity [47,48]. Our study found that *W. cibaria* SDS2.1 alone did not cause cell damage and that pretreatment with *W. cibaria* SDS2.1 alleviated *K. pneumoniae*-induced LDH release.

Many mastitis-causing bacteria, including *K. pneumoniae*, can adhere to epithelial cells. This initial attachment is a critical first step in infection, allowing bacteria to invade host cells and potentially evade antibiotics [44]. In our previous research, we found that *K. pneumoniae* from cows with mastitis readily adheres to these cells [3]. Our study demonstrates that *W. cibaria* SDS2.1 significantly reduces *K. pneumoniae* adhesion and colonization both in vivo and in vitro (*p* < 0.05). This result aligns with other studies, indicating that probiotics such as *Lactobacillus lactis CRL1655* and *Streptococcus perolens* can effectively inhibit the adhesion and colonization of various mastitis pathogens [49]. MPO activity, a marker of neutrophil activation, directly reflects the level of neutrophil aggregation in the mammary gland [50]. The administration of *W. cibaria* SDS2.1 significantly reduced the elevated MPO activity induced by *K. pneumoniae* in mice. Histopathological examination revealed extensive inflammatory cell infiltration and necrotic epithelial tissue in the KP group, whereas supplementation with *W. cibaria* SDS2.1 significantly reduced both inflammatory cell infiltration and tissue damage. These findings indicate that *W. cibaria* SDS2.1 can alleviate *K. pneumoniae*-induced mastitis in mice.

Inflammation is the immune system’s response to harmful stimuli, such as pathogens, damaged cells, toxic compounds, or irradiation [51]. When harmful pathogens invade the udder, the immune system releases inflammatory cytokines, which play a crucial role in helping epithelial cells resist infection [52]. Adequate levels of pro-inflammatory cytokines are necessary for an effective immune response. An excessive immune response not only leads to acute inflammation but may also progress to chronic inflammation, complicating the treatment of mastitis [53]. Furthermore, understanding the balance between pro-inflammatory and anti-inflammatory cytokines is crucial for modulating immune responses, as this balance helps to avoid the occurrence of long-term or chronic inflammation, thereby effectively preventing the recurrence of mastitis. Our results demonstrate that *W. cibaria* SDS2.1 effectively reduces the release of inflammatory mediators both in vivo and in vitro, thereby alleviating *K. pneumoniae*-induced mastitis. In our previous research, we found that *K. pneumoniae* isolates adhered to and invaded bMECs, resulting in the increased transcription of genes encoding IL-1β, IL-6, IL-8, and TNF-α, as well as the increased production of IL-1β, IL-8, and TNF-α [3]. IL-1β production is triggered early in infection, acting like a danger signal to initiate the inflammatory response. The secretion of TNF-α, a multifunctional pro-inflammatory cytokine, induces the production of other pro-inflammatory factors, such as IL-6, during the inflammatory response [53]. Our study demonstrates that *W. cibaria* SDS2.1 effectively reduces the release of inflammatory cytokines both in vitro and in vivo, thereby alleviating *K. pneumoniae*-induced mastitis. This anti-inflammatory effect is supported by previous research. For instance, *Lactobacillus casei* BL23 has been shown to reduce the expression of IL-6, IL-8, IL-1β, and TNF-α in bMECs stimulated by *Staphylococcus aureus*, demonstrating its anti-inflammatory properties [54]. Similarly, *L. plantarum* KLDS 1.0344 has been found to decrease *E. coli*-induced inflammation in the mammary gland by downregulating NF-κB-mediated signaling [12]. *Lactococcus lactis* 03 has also demonstrated protective effects against *E. coli*-induced mastitis in both in vitro and in vivo models [55]. Building upon these findings, our results further show that *W. cibaria* SDS2.1 not only protects bMECs from *K. pneumoniae* infection but also significantly reduces inflammation, suggesting its potential as a therapeutic agent for bovine mastitis.

**Figure 7:**
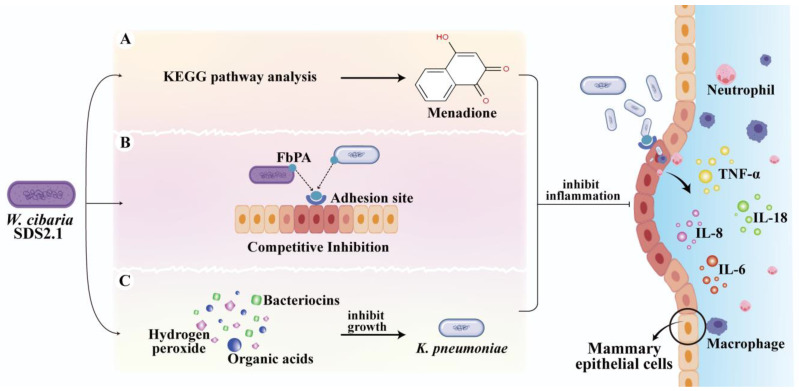
Anti-inflammatory mechanism hypothesis of *W. cibaria* SDS2.1. (**A**) Generation of anti-inflammatory metabolites by *W. cibaria* SDS2.1 (**B**) Competitive inhibition of *K. pneumoniae* adhesion by *W. cibaria* SDS2.1. (**C**) Secretion of antimicrobial metabolites by *W. cibaria* SDS2.1.

In this study, *W. cibaria* SDS2.1 significantly inhibited the adhesion of *K. pneumoniae* and alleviated inflammation in both in vitro and in vivo models. This effect may be attributed to several mechanisms, including the production of antimicrobial and anti-inflammatory metabolites and the competitive inhibition of epithelial binding sites (Figure 7). One potential mechanism for the inhibitory effect of *W. cibaria* SDS2.1 may be related to the proteins present in *W. cibaria* SDS2.1, which enable the bacterium to adhere to host cells and competitively inhibit the binding of *K. pneumoniae* to epithelial binding sites, thereby reducing the adhesion and invasion of *K. pneumoniae*. For example, the FbpA protein in *W. cibaria* has been shown to inhibit the adhesion of pathogenic bacteria [18]. Our findings also suggest that *W. cibaria* SDS2.1 can effectively reduce the adhesion and invasion of *K. pneumoniae*. Additionally, the antimicrobial effect of *W. cibaria* SDS2.1 may be attributed to specific metabolites produced by the bacterium. In this study, *W. cibaria* SDS2.1 significantly inhibited the growth of *K. pneumoniae* after co-culturing for 12 h, likely due to metabolites such as bacteriocins, organic acids, acetoin, and hydrogen peroxide, which have been closely associated with the antimicrobial properties of lactic acid bacteria [56]. Furthermore, KEGG pathway analysis of *W. cibaria* SDS2.1 revealed that it is involved in several anti-inflammatory and antimicrobial-related pathways, such as the ubiquinone and other terpenoid-quinone biosynthesis pathway. This pathway primarily synthesizes coenzyme Q and other terpenoid-quinones, such as vitamin K. In this pathway, menadione (Vitamin K3), which is synthesized, has been shown to possess anti-inflammatory and antioxidant effects [57]. However, the antimicrobial and anti-inflammatory metabolites produced by *W. cibaria* SDS2.1 still require further research to fully understand their mechanisms of action. Thus, by reducing both microbial growth and inflammation, *W. cibaria* SDS2.1 offers a dual-action approach to alleviating mastitis, which could lead to healthier dairy cows and more efficient milk production.

Although the mouse model provided valuable data for evaluating the alleviating effects of *W. cibaria* SDS2.1 on *K. pneumoniae*-induced mastitis, the generalizability of the results is somewhat limited due to the physiological and immune system differences between mice and cows. Therefore, future studies should validate these findings in bovine models. Additionally, while this study has validated the antimicrobial and anti-inflammatory functions of *W. cibaria* SDS2.1, its exact mechanisms of action and the production of metabolites still require further investigation. Overall, this study provides valuable insights into the potential application of *W. cibaria* SDS2.1 in preventing or treating bovine mastitis, but future mechanistic studies are essential to fully understand the therapeutic potential of this probiotic.

## 5. Conclusions

Our findings indicate that *W. cibaria* SDS2.1 shows strong potential as an alternative treatment for *K. pneumoniae*-induced bovine mastitis. It effectively inhibits *K. pneumoniae* growth, reduces bacterial adhesion and invasion, and alleviates inflammation through the downregulation of key inflammatory cytokines. These results provide a strong foundation for further research into *W. cibaria* SDS2.1 as an alternative therapy for mastitis, offering a potential solution to managing this condition. Future research should focus on identifying the antimicrobial substances secreted by *W. cibaria* SDS2.1—exploring their molecular mechanisms—and evaluating its potential to preserve lactation function in dairy cows, thus promoting the broader application of probiotic therapies in the dairy industry.

## Figures and Tables

**Table 1:** Experimental conditions and concentrations for antibacterial assay.

Group	Treatment Condition	Times (h)	Experimental Conditions
KP	*K. pneumoniae* (1 × 10^9^ CFU/mL)	0	The solutions were incubated at 37 °C, 180 r/min, and the viable count of *K. pneumoniae* at each time point was measured using the plate count method.
		12	
		24	
KP + SDS2.1	*K. pneumoniae* + *W. cibaria* SDS2.1 (1 × 10^9^ CFU/mL)	0	
		12	
		24	

**Table 2:** Overall condition of mouse clinical score.

Clinical Score	Criteria
1	Active and healthy
2	Slower reaction, otherwise healthy
3	Lethargic, but active
4	Inactive, but responsive
5	Unresponsive to stimuli

**Table 3:** List of primers for real-time PCR.

Gene	Primer Sequence (5′-3′)		Size
Bos			
IL-6	Forward	GCTGAATCTTCCAAAAATGGAGG	215
	Reverse	GCTTCAGGATCTGGATCAGTG	
IL-8	Forward	ACACATTCCACACCTTTCCAC	149
	Reverse	ACCTTCTGCACCCACTTTTC	
IL-1β	Forward	CCTCGGTTCCATGGGAGATG	119
	Reverse	AGGCACTGTTCCTCAGCTTC	
TNF-α	Forward	TCCAGAAGTTGCTTGTGCCT	144
	Reverse	CAGAGGGCTGTTGATGGAGG	
IL-18	Forward	TTGCATCAGCTTTGTGGAAA	78
	Reverse	TGGGGTGCATTATCTGAACA	
GAPDH	Forward	GTCTTCACTACCATGGAGAAGG	201
	Reverse	TCATGGATGACCTTGGCCAG	
Mice			
IL-1β	Forward	CCTGGGCTGTCCTGATGAGAG	188
	Reverse	TCCACGGGAAAGACACAGGTA	
IL-6	Forward	TAGTCCTTCCTACCCCAATTTCC	142
	Reverse	TTGGTCCTTAGCCACTCCTTC	
TNF-α	Forward	CAGGCGGTGCCTATGTCTC	155
	Reverse	CGATCACCCCGAAGTTCAGTAG	
GAPDH	Forward	AGGTCGGTGTGAACGGATTTG	139
	Reverse	TGTAGACCATGTAGTTGAGGTCA	

Cycle threshold (Ct) values were determined and relative mRNA expression was calculated using the comparative Ct method [34].

## Data Availability

Data are contained within the article.

## Supplementary Materials

The following supporting information can be downloaded at: https://www.mdpi.com/article/10.3390/ani15081139/s1, Figure S1: Circular map of the chromosome of *W. cibaria* SDS2.1; Figure S2: KEGG pathway classification statistical bar chart and GO annotation classification of *W. cibaria* SDS2.1 genes; Figure S3: EggNOG classification statistical bar chart and CAZy annotation of *W. cibaria* SDS2.1; Figure S4: The safety and antibacterial activity of *W. cibaria* SDS2.1; Figure S5. The VirulenceFinder analysis result of *W. cibaria* SDS2.1; Table S1: The KEGG pathway annotations table; Table S2: The card databases analysis result of *W. cibaria* SDS2.1.

## Abbreviations

The following abbreviations are used in this manuscript:

*K. pneumoniae*	*Klebsiella pneumoniae*
bMECs	bovine mammary epithelial cells
*E. coli*	*Escherichia coli*
LAB	lactic acid bacteria
*W. cibaria*	*Weissella cibaria*
*S. aureus*	*Staphylococcus aureus*
MRS	de Man Rogosa Sharp
CFU	colony-forming unit
rRNA	ribosomal RNA
tRNA	transfer RNA
GO	Gene Ontology
KEGG	Kyoto Encyclopedia of Genes and Genomes
CARD	Comprehensive Antibiotic Resistance Database
CAZy	Carbohydrate-Active enZymes Database
MICs	minimal inhibitory concentrations
MCOFFs	microbiological cut-off values
EFSA	European Food Safety Agency
TSA	Tryptic Soy Agar
MCIC	MacConkey inositol adonitol carbenicillin agar
MHA	Mueller–Hinton agar
MOI	multiplicity of infection
FBS	fetal bovine serum
SPF	Specific Pathogen Free
PBS	phosphate-buffered saline
H&E	hematoxylin and eosin
MPO	myeloperoxidase
IL	Interleukin
TNF	Tumor Necrosis Factor
GAPDH	glyceraldehyde-3-phosphate dehydrogenase
Ct	cycle threshold
LDH	lactate dehydrogenase
SEMs	standard errors of means
AMR	antimicrobial resistance
WGS	whole genome sequencing
