# Supplementary material for: Anti-Inflammatory Effects of Weissella cibaria SDS2.1 Against Klebsiella pneumoniae-Induced Mammary Gland Inflammation
Source: Animals (Basel). 2025 Apr 15;15(8):1139. doi: 10.3390/ani15081139 (PMC12024108; doi:10.3390/ani15081139)
Supplement: Supplementary file 1 [file animals-15-01139-s001.zip › animals-3538193-supplementary.pdf]

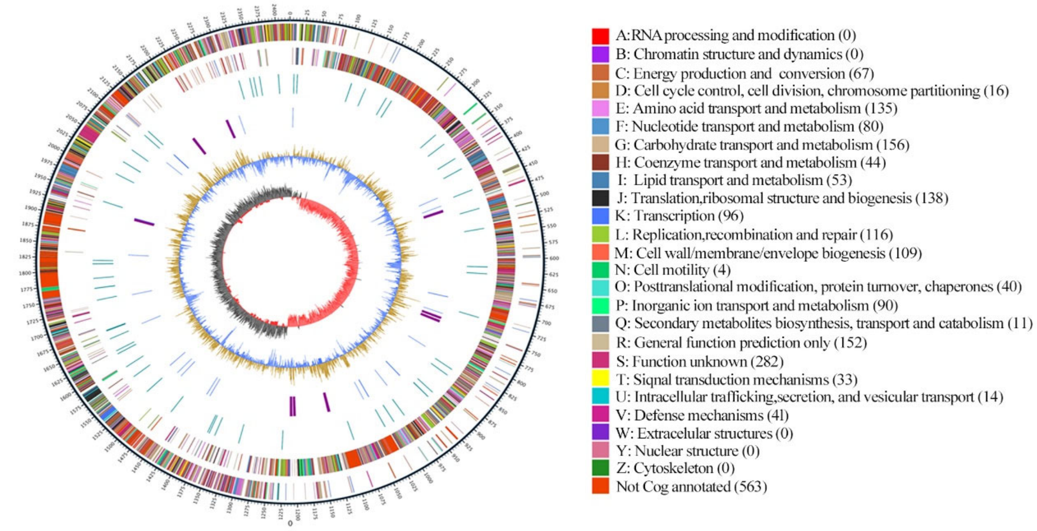

Figure S1. Circular map of the chromosome of W. cibaria SDS2.1. The outermost circle represents the genome size, with each scale corresponding to 5 kb. The second and third circles display genes on the positive and negative strands of the genome, with different colors indicating various COG functional categories. The fourth circle highlights repetitive sequences within the genome. The fifth circle shows tRNA and rRNA genes, where tRNA is colored blue and rRNA is colored purple. The sixth circle illustrates the GC content, with light yellow regions indicating areas with a higher-than-average GC content, and blue regions representing areas with a lower-than-average GC content. The innermost circle shows the GC-skew, where dark gray indicates regions with a higher G-content than C, and red indicates areas where the C-content exceeds that of G.

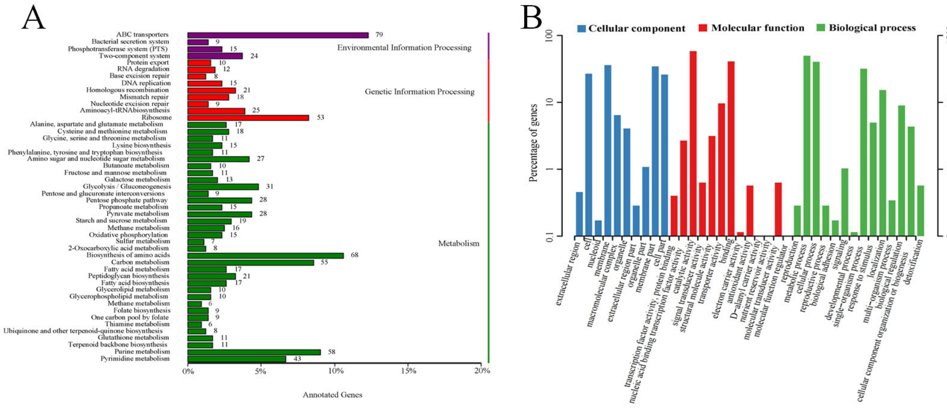

Figure S2. KEGG pathway classification statistical bar chart and GO annotation classification of *W. cibaria* SDS2.1 genes. (A) KEGG pathway classification statistical bar chart of *W. cibaria* SDS2.1 genes, illustrating the functional roles of the genes according to the KEGG pathway. (B) GO annotation classification of *W. cibaria* SDS2.1 genes, with blue representing cellular components, red representing molecular functions, and green representing biological processes

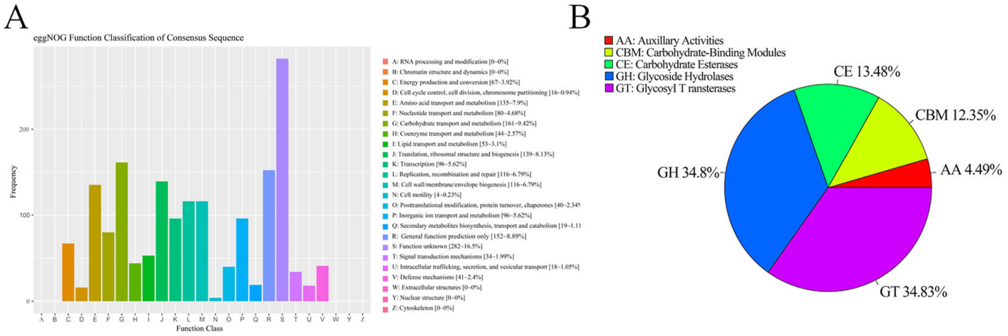

Figure S3. EggNOG classification statistical bar chart and CAZy annotation of *W. cibaria* SDS2.1. (A) EggNOG classification statistical bar chart, showing the distribution of genes into functional categories. (B) CAZy annotation of *W. cibaria* SDS2.1, displaying carbohydrate-active enzymes.

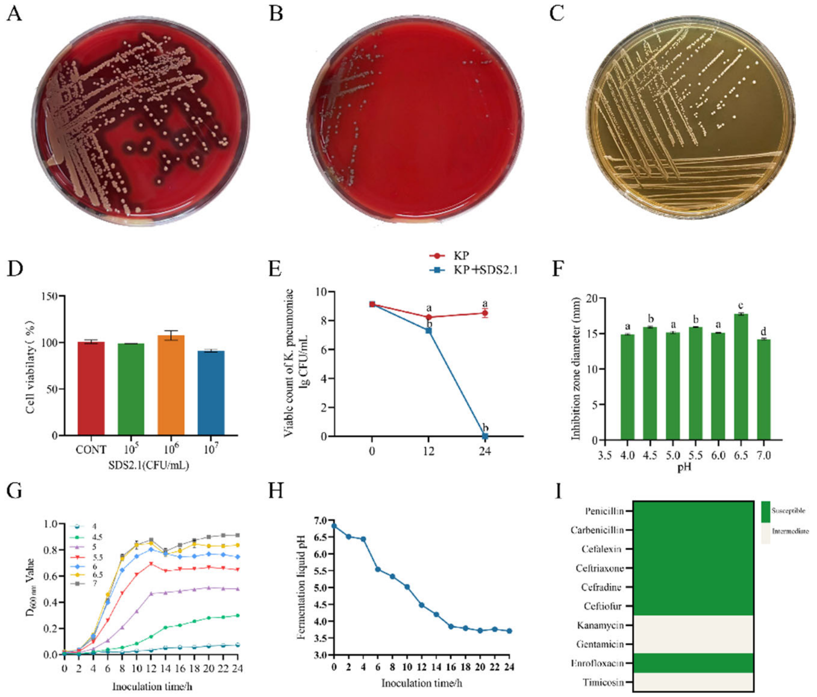

Figure S4. The safety and antibacterial activity of *W. cibaria* SDS2.1. (**A, B**) Hemolytic test of *S. aureus* and *W. cibaria* SDS2.1. (C) Morphology of *W. cibaria* SDS2.1 on MRS agar medium. (D) Cytotoxicity test of *W. cibaria* SDS2.1. (E) Co-culture growth inhibition of *K. pneumoniae*. *K. pneumoniae* and *W. cibaria* SDS2.1 were inoculated at an initial concentration of 1×10^9 CFU/mL (F) Antibacterial effects of *W. cibaria* SDS2.1 under different pH conditions. (G) Growth curves of *W. cibaria* SDS2.1 under different pH conditions. (H) Acid production curve of *W. cibaria* SDS2.1. (I) Sensitivity of *W. cibaria* SDS2.1 to antibiotics. The green color indicates susceptibility, while the off-white color represents intermediate resistance. The antibiotics tested include Penicillin, Carbenicillin, Cefalexin, Ceftriaxone, Cefradine, Ceftiofur, Kanamycin, Gentamicin, Enrofloxacin, and Tilmicosin. Data presented as mean ± SEM for three independent experiments. Different letters (a, b, c, d) indicate significant differences ($p < 0.05$) between treatments.

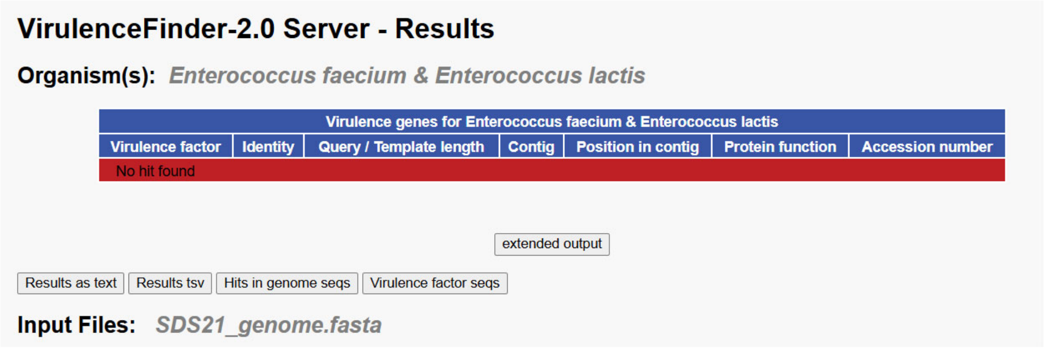

Figure S5. The VirulenceFinder analysis result of *W. cibaria* SDS2.1.

Table S1. The KEGG pathway annotations table

No	Pathway ID	Description	Gene Number
1	ko00061	Fatty acid biosynthesis	17
2	ko00130	Ubiquinone and other terpenoid-quinone biosynthesis	8
3	ko00190	Oxidative phosphorylation	15
4	ko00220	Arginine biosynthesis	11
5	ko00230	Purine metabolism	58
6	ko00240	Pyrimidine metabolism	43
7	ko00261	Monobactam biosynthesis	4
8	ko00330	Arginine and proline metabolism	5
9	ko00362	Benzoate degradation	2
10	ko00430	Taurine and hypotaurine metabolism	3
11	ko00521	Streptomycin biosynthesis	3
12	ko00550	Peptidoglycan biosynthesis	21
13	ko00590	Arachidonic acid metabolism	1
14	ko00650	Butanoate metabolism	10
15	ko00730	Thiamine metabolism	6
16	ko00740	Riboflavin metabolism	6
17	ko00760	Nicotinate and nicotinamide metabolism	6
18	ko00900	Terpenoid backbone biosynthesis	11

Table S2. The card databases analysis result of *W. cibaria* SDS2.1.

Gene	Length (bp)	Resistance Mechanism	AMR Gene Function	% Identity of Matching Region
vanT gene in vanG cluster	960	antibiotic target alteration	glycopeptide resistance gene cluster, vanT	33.33
vanY gene in vanB cluster	556	antibiotic target alteration	glycopeptide resistance gene cluster, vanY	34.95
